# The Antioxidant and Neuroprotective Potential of Leaves and Inflorescences Extracts of Selected Hemp Varieties Obtained with scCO_2_

**DOI:** 10.3390/antiox12101827

**Published:** 2023-10-04

**Authors:** Anna Stasiłowicz-Krzemień, Szymon Sip, Piotr Szulc, Jarosław Walkowiak, Judyta Cielecka-Piontek

**Affiliations:** 1 Department of Pharmacognosy and Biomaterials, Faculty of Pharmacy, Poznan University of Medical Sciences, Rokietnicka 3, 60-806 Poznan, Poland; szymonsip@ump.edu.pl; 2 Department of Agronomy, Poznań University of Life Sciences, Dojazd 11, 60-632 Poznan, Poland; piotr.szulc@up.poznan.pl; 3 Department of Pediatric Gastroenterology and Metabolic Diseases, Poznan University of Medical Sciences, Szpitalna 27/33, 60-572 Poznan, Poland; jarwalk@ump.edu.pl; 4 Department of Pharmacology and Phytochemistry, Institute of Natural Fibres and Medicinal Plants, Wojska Polskiego 71b, 60-630 Poznan, Poland

**Keywords:** *Cannabis sativa*, antioxidant, neuroprotection

## Abstract

*Cannabis sativa*, a versatile plant with numerous varieties, holds promising potential for a wide range of biological activity. As raw materials for research, we chose leaves and inflorescences of hemp varieties such as Białobrzeskie, Henola, and Tygra, which are cultivated mainly for their fibers or seeds. The choice of extraction is a key step in obtaining the selected compositions of active compounds from plant material. Bearing in mind the lipophilic nature of cannabinoids, we performed supercritical carbon dioxide (scCO_2_) extraction at 50 °C under 2000 (a) and 6000 PSI (b). The cannabinoid contents were determined with the use of the HPLC-DAD method. The antioxidant capabilities were assessed through a series of procedures, including the DPPH, ABTS, CUPRAC, and FRAP methods. The capacity to inhibit enzymes that play a role in the progression of neurodegenerative diseases, such as acetylcholinesterase (AChE), butyrylcholinesterase (BChE), and tyrosinase was also studied. The dominant cannabinoids in the extracts were cannabidiol (CBD) and cannabidiolic acid (CBDA). The highest concentration of eight cannabinoids was detected in the Tygra inflorescences extract (b). The most notable antioxidant properties were provided by the Tygra inflorescences extract (b). Nonetheless, it was the Henola inflorescences extract (b) that demonstrated the most efficient inhibition of AChE and BChE, and tyrosinase was inhibited the most significantly by the Białobrzeskie inflorescences extract (b). Multidimensional comparative analysis enrolled all assays and revealed that the Henola inflorescences extract (b) showed the most substantial neuroprotective potential.

## 1. Introduction

Cannabis plant material is essential for the development of many economic areas. For instance, the value of the European CBD market in 2020 was estimated at USD 1.7–1.9 billion [1,2]. According to many projections, it is anticipated that CBD sales in international markets will be rising within the next few years [2,3,4,5].

Among the most dynamically evolving results featuring *Cannabis* sp. include the development of dietary supplements and medicines containing cannabinoids (mainly cannabidiol (CBD) and Δ^9^-tetrahydrocannabinol (THC)). Cannabinoids are synthesized in the cannabis plant through a complex series of biochemical reactions from cannabigerolic acid (CBGA) and cannabigerovarinic acid (CBGVA) which originate from olivetolic and divarinic acids [6,7]. CBGA can be converted into tetrahydrocannabinolic acid (THCA) and cannabidiolic acid (CBDA) [8]. Cannabinoids are stored in carboxylic acid forms, as fresh plants generally do not exhibit significant concentrations of neutral cannabinoids [7,9]. These acidic cannabinoids undergo nonenzymatic decarboxylation and conversion to non-acidic forms (THC, CBD) when exposed to light, heat, or combustion during drying, heating, or smoking [7,10]. The cannabinoids present in dried cannabis plant material such as CBD, THC, cannabigerol (CBG), cannabinol (CBN), and cannabichromene (CBC) have significant potential for clinical use. 

The confirmed pharmacological activity of cannabinoids includes reducing muscle spasticity and spasms in patients with multiple sclerosis [11], reducing neuropathic pain [12], migraines [13], reducing the number of seizures in epilepsy [14,15], relieving the effects of irritable bowel syndrome [16], Crohn’s disease symptoms [17], depression [18], and reducing anxiety and its related symptoms (such as insomnia) [19,20]. Recent scientific reports indicate a possibility of alleviating the pain associated with endometriosis [21], and vulvodynia [22], and enhancing the quality of life for patients and to enable them to engage more effectively with their surroundings. [23].

One of the essential steps in the extraction of cannabinoids is their isolation from the plant matrix. Cannabinoid derivatives are characterized by their lipophilic nature, which limits their water solubility, thus there is a need to use organic solvents [24,25,26,27]. However, the use of organic solvents comes with notable drawbacks, not only for human health but also for the environment [28,29,30,31,32]. The dynamics of the hemp market necessitate the exploration and implementation of green, health-conscious alternatives that are eco-friendly instead of organic solvents during cannabis processing steps. Among the many extraction techniques of cannabinoids are maceration, dynamic maceration, soxhlet extraction, ultrasound-assisted extraction, microwave-assisted extraction, subcritical fluid extraction, and supercritical fluid extraction [33,34,35]. The use of supercritical fluid extraction (SFE) with the application of CO_2_ has been consistently extended to the extraction of a set of cannabinoids (different levels) from different varieties of *Cannabis* sp. [36,37] without the production of organic waste and without the need to control their residues in the extracts. Different varieties of cannabis plants might show different profiles of secondary plant metabolites and, as a result, their biological activity [38,39]. 

The inspiration for this study was the confirmation of the antioxidant potential of leaves of the fibrous hemp varieties Tygra and Białobrzeskie, and Henola (grown mainly for oil), which until now have been rarely used as biologically active systems [40,41]. All three varieties are listed in the European Union Plant variety database and have been approved by the Colorado Department of Agriculture, USA [40]. In our earlier work, we collected the results of research that confirmed the antioxidant potential of the leaves of selected hemp varieties (Tygra, Białobrzeskie, Henola) [42]. Bearing in mind the biological properties of the obtained cannabinoid compositions, we began to look for more environmentally friendly methods of obtaining them. By choosing the supercritical carbon dioxide (scCO_2_) extraction technique, we met the criteria for using a green approach to the processing of plant raw materials. To the best of our knowledge, the results of scCO_2_ extraction of cannabinoid profiles present in hemp fiber varieties such as Białobrzeskie, Tygra, and Henola are published for the first time in the field of application of this extraction technique, evaluation of the composition of the extracts obtained in this approach, as well as evaluation of the biological properties of the extracted cannabinoid compositions.

With regard to the above, the aim of our research was to obtain extracts from the leaves and inflorescences of Białobrzeskie, Tygra, and Henola hemp varieties by scCO_2_ extraction and to evaluate their neuroprotective properties.

## 2. Materials and Methods

### 2.1. Plant Materials

The plant material for analysis—Białobrzeskie, Tygra, and Henola varieties—were generously provided by the Experimental Station for the Cultivar Testing in Chrząstowo, belonging to the Research Centre for Cultivar Testing in Słupia Wielka. In 2022, sugar beet served as the preceding crop for hemp cultivation. Individual tillage operations were carried out following agrotechnical recommendations for this species (winter plowing 29 October 2021; 17 March 2022 harrow + spear, 6 May 2022 cultivation unit; 9 May 2022 sowing). The day after the sowing of hemp (10 May 2022), Boxer 800 EC herbicide was applied at a rate of 2.6 l/ha. Mineral fertilization was conducted based on the following mineral fertilizers: Lubofos 12 (200 kg/ha), potassium salt (183 kg/ha), enriched superphosphate (115 kg/ha), urea (159 kg/ha), and salmag (119 kg/ha). The soil of the experimental field was classified as IIIa, complex 2. Concerning grain size, the top horizons of the examined soil were categorized as loamy sands, with a clay fraction comprising 4%, dust 14%, and sand fraction 83%. The eluvial level contained slightly less clay fraction and dust fraction. The enrichment (B) and bedrock levels were more compact. The pH measured in the aqueous extract expressed in pH units was 6.80, while in KCl it was about 0.5 units lower and fell within the upper values of the slightly acidic range. The organic carbon content was about 1%, which, in terms of humus, is 1.7%. The total nitrogen content was assessed as 0.086% and the C:N ratio was about 12:1. Thermal and moisture conditions during the growing season were favorable for cannabis growth and development. The collection of plant material for the study occurred once the hemp plants had reached the maturation phase, i.e., from the moment of seed formation to the first seed. Following collection, two separate 500 g samples were set aside and subjected to drying until they reached a dry state. This drying process spanned a total of twenty hours. During the initial six hours, the oven temperature was carefully controlled, not exceeding 50 °C, while for the subsequent 14 h, the oven was kept at a constant temperature of 105 °C.

### 2.2. Reagents

Cannabinoids standards: CBD, CBDA, Δ^9^-THC, THCA, CBG, CBGA, CBN, and CBC were obtained from Sigma-Aldrich (Poznan, Poland). Trifluoroacetic acid and acetonitrile (high-performance liquid chromatography [HPLC] grade) were supplied by Merck (Darmstadt, Germany). High-quality pure water was prepared using a Direct-Q 3 UV purification system (Millipore, Molsheim, France; model Exil SA 67120). Iron (III) chloride hexahydrate, 5,5-dithio-bis-(2-nitrobenzoic acid), 2,2-diphenyl-1-picrylhydrazyl, 2,2′-azino-bis(3-ethylbenzothiazoline-6-sulfonic acid), neocuproine, 2,4,6-tri(2-pyridyl)-s-triazine, trolox, tyrosinase, L-DOPA, acetylcholinesterase, butyrylcholinesterase, acetylcholine iodide, butyrylcholine iodide, Trizma^®^ hydrochloride, and Trizma^®^ base were purchased from Sigma-Aldrich (Schnelldorf, Germany). Sodium chloride, sodium dihydrogen phosphate, and sodium hydrogen phosphate, were purchased from Avantor Performance Materials (Gliwice, Poland). Ammonium acetate (NH_4_Ac) and methanol were supplied by Chempur (Piekary Śląskie, Poland). Cupric chloride dihydrate, acetic acid (99.5%), ethanol (96%), and sodium acetate trihydrate were supplied by POCH (Gliwice, Poland). 

### 2.3. Extraction

Dry raw leaves and inflorescences of Białobrzeskie, Tygra, and Henola varieties were ground and 6.5 g of the plant material was loaded into an extraction vessel. The dynamic scCO_2_ extraction process was carried out under 2000 and 6000 PSI at 50 °C with 250 mL of CO_2_ as marginal pressure values significantly affecting the neuroprotective activity and exhaustive amount of carbon dioxide assessed during preliminary studies (Table 1). Next, the extracts were suspended in methanol, winterized, and filtered under vacuum.

### 2.4. Chromatographic Analysis

The cannabinoid profile analysis of the extract was studied with the use of ultra-high-performance liquid chromatography with the diode array detector (HPLC-DAD) validated method (Shimadzu Corp., Kyoto, Japan) [42]. The determination was performed with the use of a chromatographic column CORTECS Shield RP18, 2.7 µm; 150 mm × 4.6 mm. As a mobile phase, 0.1% trifluoroacetic acid (41%), and acetonitrile (41:59, *v*/*v*) were used. The flow rate was set at 2.0 mL/min, and the column temperature was set at 35 °C. The injection volume was 10.0 µL, the detection wavelength was 228 nm, and the analysis time was 50 min. The retention time of cannabinoids is presented in Table 2. The results were acquired and processed using LabSolutions LC software (version 1.86 SP2) from Shimadzu Corp. (Kyoto, Japan).

### 2.5. Antioxidant Activity

The antioxidant activity was performed with the use of four assays: DPPH, ABTS, CUPRAC, and FRAP. Each assay was preceded by the screening of the extracts’ antioxidant activity with descending concentrations of the extracts. Trolox antioxidant activity was studied at a suitable concentration range to inhibit radicals (DPPH and ABTS) or to perform redox reactions (CUPRAC and FRAP). A linear regression equation was established to relate the trolox concentration with its corresponding scavenging percentage (DPPH and ABTS) or absorbance (CUPRAC and FRAP), subsequently, the results presented as mg trolox/g plant material were calculated through the equation, according to the antioxidant properties of the extracts in all four assays [43,44]. The antioxidant potential was determined according to the previously reported method [45,46]. Each measurement in the antioxidant studies was repeated six times.

The DPPH assay is based on mixing the radical solution with a hydrogen atom-donating substance, as it causes the vibrant violet color to vanish, leading to the formation of the reduced form [47]. The inhibition of DPPH radicals by the studied samples/trolox was calculated using the formula:

(1) $$DPPH\;\;scavenging\;\;activity(\%)\frac{A_{o}-A_{i}}{A_{o}}\times 100\%$$

where *A_o_* is the absorbance of the control sample and *A_i_* is the absorbance of the test sample. 

The other measurement that determined the scavenging radicals potential was the ABTS assay in which green cation radicals are produced by the loss of electrons by the nitrogen atoms of ABTS, caused by potassium persulfate. The inhibition of ABTS•+ was calculated using the following formula:

(2) $$\mathrm{ABTS}\;\mathrm{scavenging}\;\mathrm{activity}\;(\%)=\frac{A_{0}-A_{1}}{A_{0}}\times 100\%$$

where:

*A*_0_—the absorbance of the control;

*A*_1_—the absorbance of the sample.

As a first method of determining the reducing potential of an oxidant, CUPRAC was performed. During this assay, phenolic groups of antioxidants are oxidized to quinones, and the neocuproine and copper (II) ion complex (bluish) is reduced to the neocuproine and copper (I) ion complex (yellow). 

The last antioxidant technique that determined the reducing properties (of colorless Fe^3+^ ion to Fe^2+^ with the formation of a dark blue complex with 2,4,6-tris(2-pyridyl)-1,3,5-triazine (TPTZ)) by the extracts was the FRAP technique. 

### 2.6. Inhibition of Enzymes Influencing the Neurodegenerative Diseases 

The potential of the extracts to inhibit enzymes such as AChE, BChE, and tyrosinase involved in neurodegeneration development was studied. Firstly, the screening of the extracts’ activity by measuring the inhibitory activity of the descending concentrations was performed. Esterases’ strong inhibitors are rivastigmine, donepezil, and galantamine [48], whereas tyrosinase is strongly inhibited by hydroquinone, kojic acid, and azelaic acid [49]. Thus, galantamine was chosen as a standard inhibitor for AChE and BChE, whilst for tyrosinase inhibitor, azelaic acid was selected. A linear regression equation between the standard concentration and its percentage potential to inhibit the enzyme was built, and the standard equivalent was calculated through the equation, according to the inhibitory properties of the extracts in all three assays. The results were presented as a galantamine equivalent (GALAE) (mg galantamine/g plant material) for the AChe and BChE assays and as an azelaic acid equivalent (AzAE) (mg azelaic acid/g plant material) [50,51,52,53,54,55]. 

The ability to inhibit AChE and BChE was determined according to the previously reported method [45]. This assay requires artificial substrates (thiocholine esters). Thiocholine is liberated during the enzymatic reactions with 5,5′-dithio-bis-(2-nitrobenzoic) acid (DTNB), and the 3-carboxy-4-nitrothiolate anion (TNB anion) is formed. The potential to inhibit AChE and BChe was measured according to the increase in the thiocholine color in a 96-well plate. The percentage of AChE and BChE inhibition by the samples was calculated according to the following equation:

(3) $$\mathrm{AChE}/\mathrm{BChE}\;\mathrm{inhibition}\;(\%)=\frac{1-(A_{1}-A_{1b})}{\left(A_{0}-A_{0b}\right)}\times 100\%$$

where:

*A*_1_—the absorbance of the test sample;

*A*_1*b*_—the absorbance of the blank of the test sample;

*A*_0_—the absorbance of control;

*A*_0*b*_—the absorbance of the blank of control.

The tyrosinase inhibition assay is based on the reduction in color intensity of the solution due to the inhibition of enzyme activity [56]. The inhibitor blocks L-DOPA access to the tyrosinase active site, which prevents the reaction from proceeding. The study was performed according to the previously reported method [45]. The percentage inhibition of the tyrosinase by the samples was calculated with the use of the following equation:

(4) $$\mathrm{Tyrosinase}\;\mathrm{inhibition}\;(\%)=\frac{1-(A_{1}-A_{1b})}{\left(A_{0}-A_{0b}\right)}\times 100\%$$

where:

*A*_1_—the absorbance of the test sample;

*A*_1*b*_—the absorbance of the blank of the test sample;

*A*_0_—the absorbance of control;

*A*_0*b*_—the absorbance of the blank of control.

### 2.7. Analysis of the Results

The statistical analysis was conducted using Statistica 13.3 software (StatSoft Poland, Krakow, Poland). The data is presented in the form of mean values along with their corresponding standard deviations. The experimental data were analyzed using the skewness and kurtosis tests to determine the normality of each distribution, while Levene’s test was used to assess the equality of variances [57,58]. To establish statistical significance, a one-way analysis of variance (ANOVA) was employed, and subsequently, the Bonferroni post hoc test was conducted to compare the experimental outcomes for each extract. Statistical significance was established for differences at a significance level of *p* < 0.05. We conducted Principal Component Analysis (PCA) to elucidate and interpret the interrelationships between the compound profiles and their influence on the biological activity of the extracts. This analysis was carried out using PQStat v.1.8.4.140 software (Poznań, Poland). The Pearson matrix was also calculated with PQStat software v.1.8.4.140. To identify the extract exhibiting the most robust potential neuroprotective activity, which will include both antioxidant activity (DPPH, ABTS, CUPRAC, FRAP methods) and the capacity to inhibit AChE, BChE, and tyrosinase enzymes, a multidimensional comparative analysis (MCA), which compares multi-feature objects, was performed [59,60]. Synthetic indicators are the main criterion for organizing the examined results and their ranking with the use of multidimensional comparative analysis. In the process of normalization, the considered diagnostic features were assigned a specific meaning for the assessment of objects. Standardization was used for the normalization of variables. Synthetic measures were calculated, and the rankings of regions were prepared. 

## 3. Results and Discussion

### 3.1. Extraction

Hemp varieties like Białobrzeskie, Henola, and Tygra are primarily cultivated for their seeds and fibers, contributing significantly to various industries. However, a noteworthy aspect often overlooked is the potential use of their leaves and flowers for disease prevention, given their studied and hoped-for biological activity. These plant materials, often treated as waste during the production of seeds and fibers, might possess disease-preventative potential if they possess biological activity. For example, it would be beneficial to present their antioxidant and neuroprotective activity as *Cannabis sativa* is known to have such properties [61,62,63,64]. Given the increasing focus on natural remedies and plant-based therapies, unlocking the medicinal potential of these overlooked parts could offer a sustainable and holistic approach to disease prevention, bolstered by their potential biological activity [65,66,67]. Moreover, diversifying the utilization of hemp varieties aligns with the principles of efficient resource utilization and sustainability, promoting a more comprehensive and responsible agricultural model [68]. By recognizing the value inherent in the leaves and flowers of Białobrzeskie, Henola, and Tygra, and their potential biological activity, we can maximize the benefits derived from these hemp varieties, enhancing both agricultural practices and human health on multiple fronts.

scCO_2_ is a commonly used extractant in the pharmaceutical and natural products industries [69,70,71,72,73]. It is a non-toxic, non-flammable, and environmentally friendly solvent [74,75], and it can be easily removed by depressurization and evaporation [76,77,78]. The extracts were obtained with scCO_2_ at 50 °C under 2000 (a) and 6000 PSI (b) (Table 3).

### 3.2. Chromatographic Analysis

As expected, the cannabinoid profiles differed between the studied hemp strains (Białobrzeskie, Henola, Tygra) (Table 3). In addition, changes in cannabinoid profiles were also noted between the leaves and inflorescences. Each plant material had the highest content of two cannabinoids, CBD and CBDA. The leaves had lower contents of all the tested cannabinoids such as CBD, CBDA, CBG, CBN, CBGA, THC, CBC, and THCA, particularly when compared to inflorescences. Most frequently, the extraction carried out at 6000 PSI (b) led to a higher cannabinoid content than the extraction under 2000 PSI (a). The highest CBD (6248.5 ± 154.9 μg/g plant material) and CBDA (2558.0 ± 107.8 μg/g plant material) contents were obtained in an extract of Tygra inflorescences obtained under 6000 psi. The highest contents of CBG (224.8 ± 2.0 μg/g plant material), CBN (107.7 ± 5.4 μg/g plant material), CBGA (89.74 ± 4.23 μg/g plant material), THC (998.4 ± 18.8 μg/g plant material), CBC (310.2 ± 12.9 μg/g plant material), and THCA (303.0 ± 3.9 μg/g plant material) were found in the Białobrzeskie inflorescences extract obtained under 6000 PSI.

### 3.3. Antioxidant Activity

The antioxidant properties of the extracts were studied based on two mechanisms: the first mechanism scavenges free radicals: 2,2-diphenyl-1-picrylhydrazyl (DPPH), 2,2′-azino-bis(3-ethylbenzothiazoline-6-sulfonic acid (ABTS), and the second inhibit oxidation reactions by being oxidized themselves: cupric reducing antioxidant capacity (CUPRAC), and ferric reducing antioxidant power (FRAP). 

**Figure 1:**
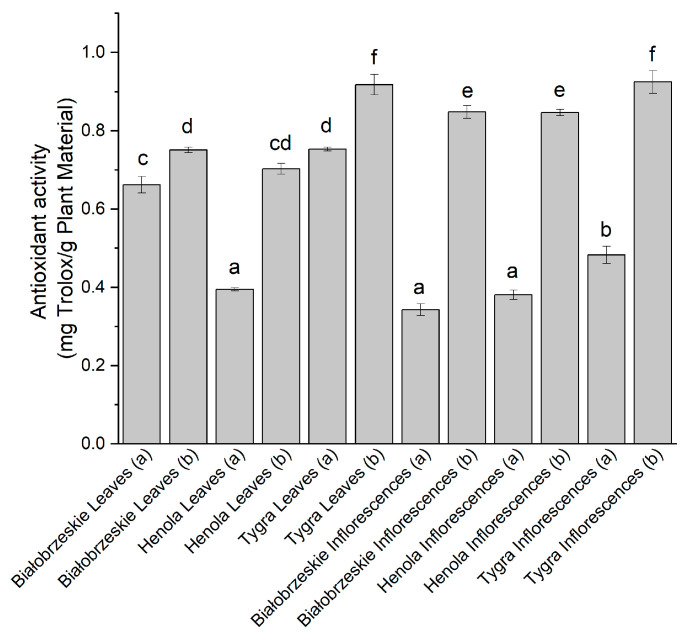
The antioxidant potential of Białobrzeskie, Henola, and Tygra leaves and inflorescences extracts obtained under 2000 PSI (a) and 6000 PSI (b), presented as mg trolox/g plant material studied in the DPPH assay. Different letters (a–f) within the bars indicate statistical differences (*p* < 0.05).

During the DPPH assay (Figure 1), the 1,1-diphenyl-2-picrylhydrazyl radical was scavenged by all the extracts at some level. The extracts obtained under 6000 PSI pressure exhibited more pronounced antioxidant activity than those obtained at the lower pressure of CO_2_. The most potent antioxidant activity against the DPPH radical was obtained by the Tygra variety, with the result 0.92 ± 0.03 mg trolox/g plant material and 0.93 ± 0.03 mg trolox/g plant material for the leaves and inflorescences, respectively. Both of the results were statistically significantly better than every other extract. 

**Figure 2:**
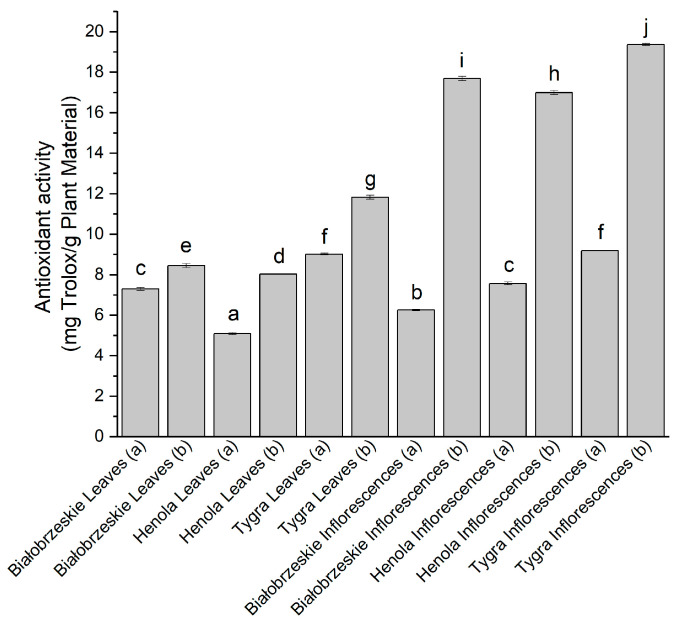
The antioxidant potential of Białobrzeskie, Henola, and Tygra leaves and inflorescences extracts obtained under 2000 PSI (a) and 6000 PSI (b), presented as mg trolox/g plant material studied in ABTS assay. Different letters (a–j) within the bars indicate statistical differences (*p* < 0.05).

Another technique that determined the radical scavenging potential of the extracts was the ABTS method (Figure 2). The ABTS•+ radical was scavenged better by the extracts obtained under 6000 PSI (b). The greatest results among cannabis leaf extracts were noticed for Tygra (b) (11.83 ± 0.10 mg trolox/g plant material). The most substantial extract was the Tygra inflorescences (b) with 19.37 ± 0.06 mg trolox/g plant material, which was significantly greater than any other extract. 

**Figure 3:**
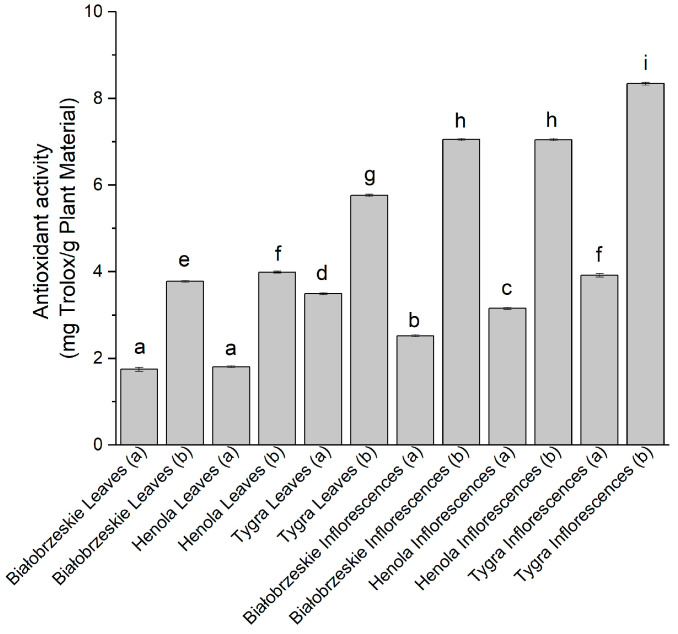
The antioxidant potential of Białobrzeskie, Henola, and Tygra leaves and inflorescences extracts obtained under 2000 PSI (a) and 6000 PSI (b), presented as mg trolox/g plant material studied in the CUPRAC assay. Different letters (a–i) within the bars indicate statistical differences (*p* < 0.05).

The ability to reduce cupric ion was also studied (CUPRAC assay) (Figure 3), and the most efficient extract among the leaves was the Tygra (b)—5.76 ± 0.01 mg trolox/g plant material, while the most noticeable potential to reduce Cu^2+^ ion was shown by Tygra in the florescences (b) extract—8.34 ± 0.01 mg trolox/g plant material, which was statistically greater than every other extract. 

**Figure 4:**
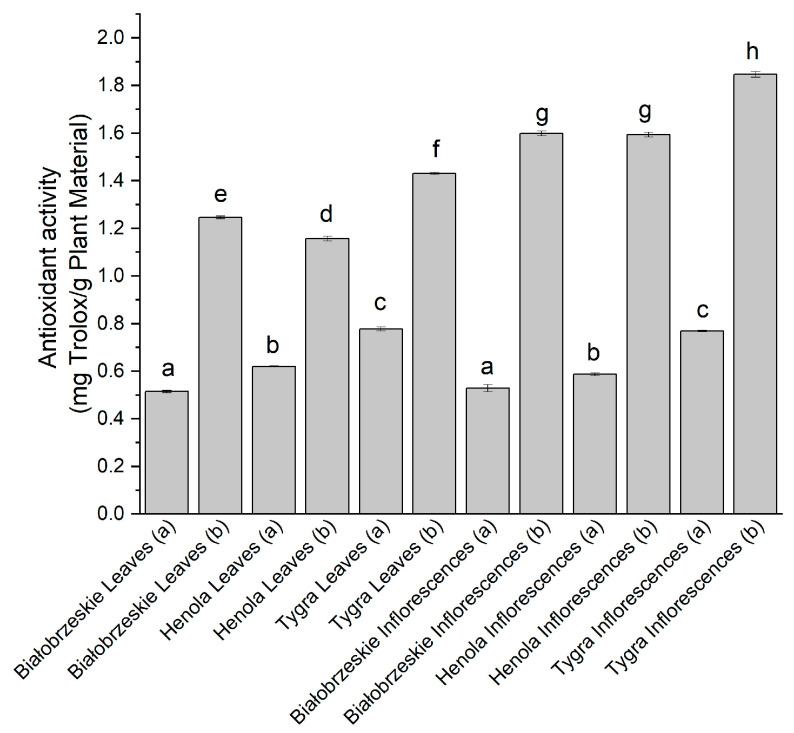
The antioxidant potential of Białobrzeskie, Henola, and Tygra leaves and inflorescences extracts obtained under 2000 PSI (a) and 6000 PSI (b), presented as mg trolox/g plant material studied in the FRAP assay. Different letters (a–h) within the bars indicate statistical differences (*p* < 0.05).

The last antioxidant model used in this study was FRAP, which determined the ability to reduce ferric ions (Figure 4). The strongest antioxidant potential within the leaves was determined for the Tygra (b)—1.43 ± 0.01 mg trolox/g plant material; whereas among inflorescences, the simultaneously the greatest result of all showed Tygra inflorescences (b) extract—1.84 ± 0.01 mg trolox/g plant material, which had a statistical significance. Overall, the most significant antioxidant activity in the four assays was noted for the Tygra variety.

The oxidative stress mechanism entails the creation of reactive oxygen species (ROS) through normal cellular metabolism, exposure to environmental toxins, or the presence of conditions that cause oxidative stress. Oxidative stress arises when there is a disturbance in the equilibrium between the generation of ROS and the body’s protective antioxidant mechanisms [79].

There are also other studies in the literature presenting the antioxidant potential of the cannabis plant. In a study by Mastellone et al., the fiber-type *Cannabis sativa* L. plant material was extracted with methanol and acetone by ultrasound-assisted extraction; subsequently, both of the extracts were tested for antioxidant properties with the use of the ABTS and DPPH assays [80]. Methanol extract showed a stronger antioxidant potential than acetone. In another study, Futura 75 hemp frozen inflorescences were extracted by microwave-assisted extraction to obtain an aqueous extract, while the residual biomass was extracted with the use of n-hexane in an ultrasound bath [62]. In the DPPH and superoxide radical uptake assays, aqueous extract presented a higher potency, while in the FRAP and ORAC assays, hexane extract was stronger. André et al. studied the influence of maturation on polyphenol content and antioxidant activity of eight different fiber-type *Cannabis sativa* L. cultivars [81]. The total phenolic content decreased with the flower’s development for all cultivars studied. Similarly, DPPH radical scavenging activity was reduced over the maturation period. The antioxidant activity of cannabis was also studied in vivo. In a study by Asta et al., the *Cannabis sativa* Futura 75 variety was extracted with ethanol and administered to the mice intragastrically (1.6 mg of cannabis extract/g/day) via stomach tube [82]. The extract contradicted the AlCl_3_-induced increase in the level of malondialdehyde in the liver and brain and prevented the decrease in catalase activity. Previous studies indicate that antioxidant activity is related to the level of polyphenols in plant material, but the level of cannabinoids contributes significantly to antioxidant activity by *Cannabis sativa*. In the present study, the most potent antioxidant activity was related to the greatest CBD level. 

In our previous research, which inspired the research presented in this paper, we confirmed the antioxidant potential of methanolic, ethanolic, and isopropanol extracts of hemp fiber varieties. The greatest antioxidant power was also associated with the highest concentrations of CBD [42]. However, it is worth mentioning that other cannabis components besides cannabinoids might also play a significant role in antioxidant properties and work synergistically with cannabinoids, which is called the “entourage” effect [2,3,4]. Different cannabis varieties show a wide range of secondary plant metabolite profiles and thus differ in biological activity [83,84]. Cannabis plant material after extraction provides compounds that are responsible for the smell, terpenes like β-myrcene, limonene, and β-(E)-caryophyllene, as well as α-humulene [35,85]. These components exert their biological activity, including antioxidant potential [64,86]. Cannabis plant material should be treated as a whole, thus it is also worth mentioning that phenolic compounds, e.g., phenolic acids and flavonoids in their glycoside and aglycone forms which are found in cannabis plant material chlorogenic and caffeic acids, catechin, epicatechin, rutin, naringenin, quercetin, apigenin, cannaflavin B and lignanamides cannabisin A, B, and C also play a significant role in cannabis antioxidant properties [64,87,88,89,90,91].

### 3.4. Anticholinesterase Activity

AChE and BChE are enzymes that break down the neurotransmitter acetylcholine in the synaptic cleft. The inhibition of these enzymes can lead to an accumulation of acetylcholine, which can enhance cholinergic neurotransmission and potentially provide neuroprotective effects. Cholinergic neurotransmission plays a crucial role in cognitive function [92], including memory and learning. In addition to the cognitive effects, AChE, and BChE inhibition may also have neuroprotective effects as they leads to a reduction in oxidative stress and inflammation, which are principal in the pathogenesis of neurodegenerative diseases [93]. Additionally, the inhibition of BChE prevents amyloid-beta-induced neuronal death [94].

**Figure 5:**
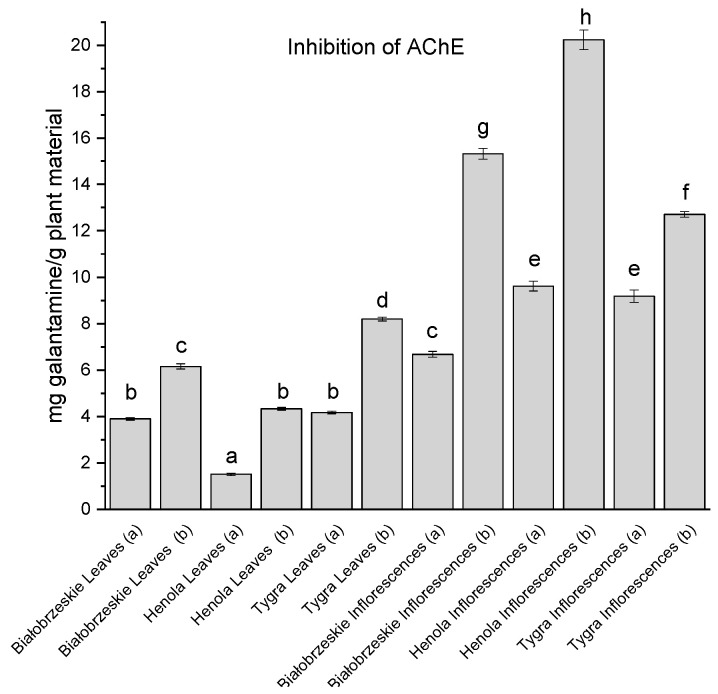
Acetylcholinesterase (AChE) inhibitory activity of Białobrzeskie, Henola, and Tygra leaves (L) and inflorescences (I) extracts obtained under 2000 PSI (a) and 6000 PSI (b) presented as mg galantamine/g plant material. The standard error is represented by the error bars. Different letters (a–h) within the bars indicate statistical differences (*p* < 0.05).

All of the leaf and inflorescence extracts showed inhibition of AChE (Figure 5). In general, the inflorescences had greater inhibitory potential than the leaves, with the greatest result noted for the Tygra leaves (b)—8.20 ± 0.08 mg galantamine/g plant material. Among the inflorescences, the most anti-acetylcholinesterase activity was noted for the Henola inflorescences (b)—20.23 ± 0.43 mg galantamine/g plant material which was statistically different than all of the studied extracts. 

**Figure 6:**
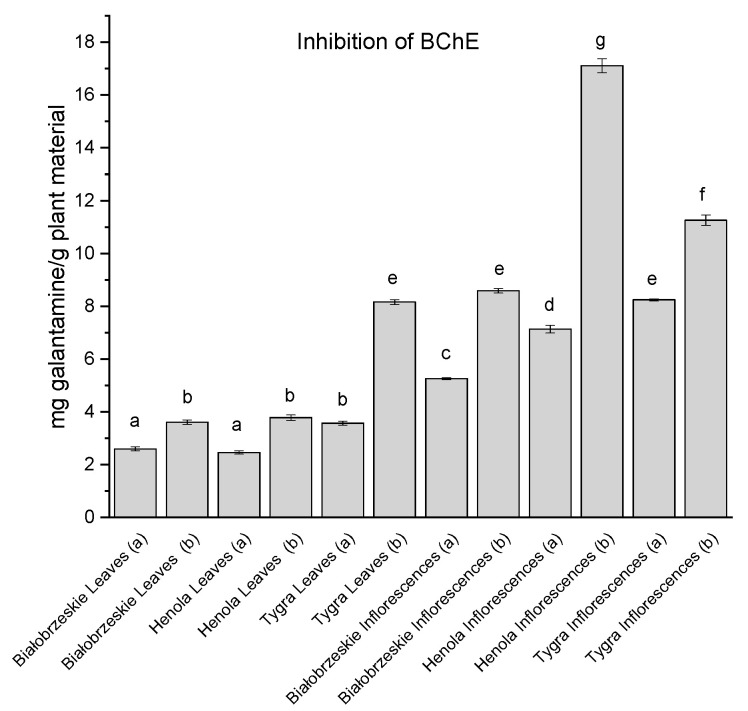
Butyrylcholinesterase (BChE) inhibitory activity of Białobrzeskie, Henola, and Tygra leaves (L) and inflorescences (I) extracts obtained under 2000 PSI (a) and 6000 PSI (b) presented as mg galantamine/g plant material. The standard error is represented by the error bars. Different letters (a–g) within the bars indicate statistical differences (*p* < 0.05).

BChE inhibitory activity was also noted within *Cannabis sativa* extracts and the overall results are similar to those of the AChE inhibition, as among the leaves the strongest inhibitory potential was assessed for the Tygra leaves (b)—8.17 ± 0.09 mg galantamine/g plant material (Figure 6). The most intense ability to inhibit AChE was determined for the Henola inflorescences (b)—17.12 ± 0.27 mg galantamine/g plant material, which has a statistically significantly stronger potential than any other studied extract.

In a study by Karimi et al., methyl alcohol *Cannabis sativa* extract derived from leaves showed inhibition of AChE and BChE activity at 52.33% and 49.00%, respectively; whereas resin fraction inhibited the AChE and BChE at 80.00% and 68.00% [95]. In their research, Puopolo et al. investigated the anticholinesterase properties of eight cannabinoids: CBD, Δ^8^-THC, CBG, CBGA, CBT, CBDV, CBC, and CBN, all at a concentration of 200 µM [96]. They observed the significant inhibition of AChE and BChE activities, with varying percentages of inhibition. Specifically, AChE was inhibited by 70.8, 83.7, 92.9, 76.7, 66.0, 79.3, 13.7, and 30.5%, while BChE was inhibited by 86.8, 80.8, 93.2, 87.1, 77.0, 78.5, 27.9, and 22.0%, respectively. Molecular docking investigations revealed that the cannabinoids engaged with multiple amino acid residues on the enzyme proteins, providing substantial evidence for their collective ability to inhibit AChE and BChE. In a study by Mooko et al., hexane and dichloromethane flower and bud extracts showed a greater inhibitory potential against AChE and BChE, whereas water, hexane, dichloromethane: methanol (1:1), and methanol showed a better potential to inhibit β-secretase [97]. In an in vivo study, where rats were administered cannabis resin, tramadol, or a combination of both, cannabis alone increased AChE brain activity (by 16.3–36.5%), while it remained at the same level after tramadol administration, whilst the combination of both decreased AChE activity (by 12.9–13.6%) [98]. The activity of BChE exhibited significant and dose-dependent inhibition in response to cannabis resin (by 60.9–76.9%), but also by tramadol (by 17.6–36.5%) and the combination of both (57.2–63.9%). In a study on *Caenorhabditis elegans,* the neuroprotective potential of two *C. sativa* oils demonstrating fluctuations in CBD and THC concentrations was studied and, as a result, both oils were efficient in decreasing AChE activity, and ROS levels [61].

### 3.5. The Inhibition of Tyrosinase

**Figure 7:**
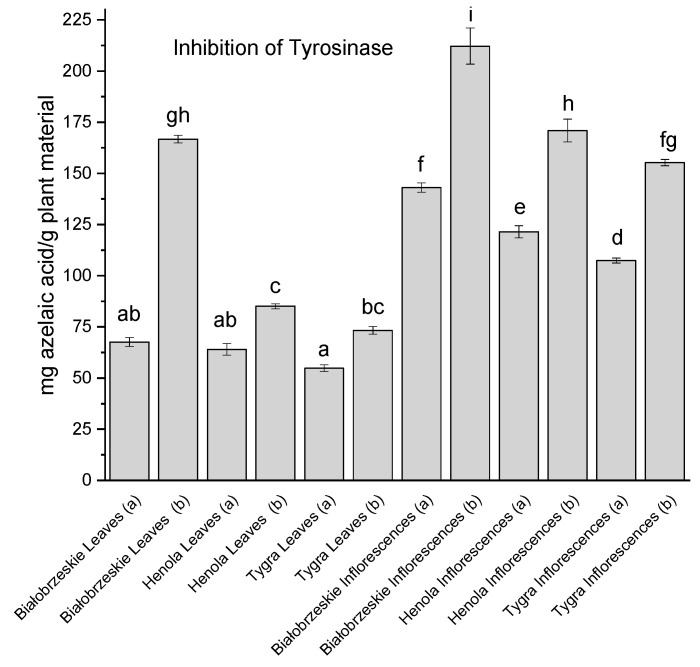
Tyrosinase inhibitory activity of Białobrzeskie, Henola, and Tygra leaves (L) and inflorescences (I) extracts obtained under 2000 PSI (a) and 6000 PSI (b) presented as mg azelaic acid/g plant material. The standard error is represented by the error bars. Different letters (a–i) within the bars indicate statistical differences (*p* < 0.05).

Tyrosinase inhibition, involving the blockage of the L-DOPA substrate access to the center of the enzyme, is a key reaction in relation to the inhibition of dopamine breakdown, which is deficient in Parkinson’s disease [99]. *Cannabis sativa* extracts inhibited tyrosinase (Figure 7).

In most cases, the leaf extracts showed weaker potential to inhibit the enzyme than the inflorescence extracts. The only exception was the Białobrzeskie leaves extract (b), which showed exceptional inhibition properties—166.73 ± 1.87 mg azelaic acid/g plant material and was not significantly different from the Henola inflorescences extract (b) 170.92 ± 5.57 mg azelaic acid/g plant material. The greatest tyrosinase inhibitor and most statistically different from all other extracts was the Białobrzeskie inflorescences extract (b)—212.22 ± 8.83 mg azelaic acid/g plant material.

In a Kim et al. study, hemp seeds ethanol crude extract was fractioned with hexane, dichloromethane, ethyl acetate, and butanol [100]. Tyrosinase’s inhibitory activity depends on the used solvents [80]. The methanol extract showed 33.17% ± 2.04% of inhibition while acetone showed 38.46% ± 1.36%. In the Manosroi et al. study, hemp leaves and seeds collected in Thailand were macerated with ethanol; subsequently, the extracts were studied for the inhibitory potential of tyrosinase [101] and they showed inhibition of the enzyme. 

It would be beneficial to extend the investigations to include experiments conducted on cell lines. After receiving promising results, it would be suggested to expand the research to in vivo animal studies to gain insights into the potential neurodegeneration-preventative applications of the extracts.

### 3.6. Statistical Analysis

PCA was used to explain the variation in the data in the present study, namely, to verify if and how the sum of the eight cannabinoids’ content impacts the biological activity of the extract. 

**Figure 8:**
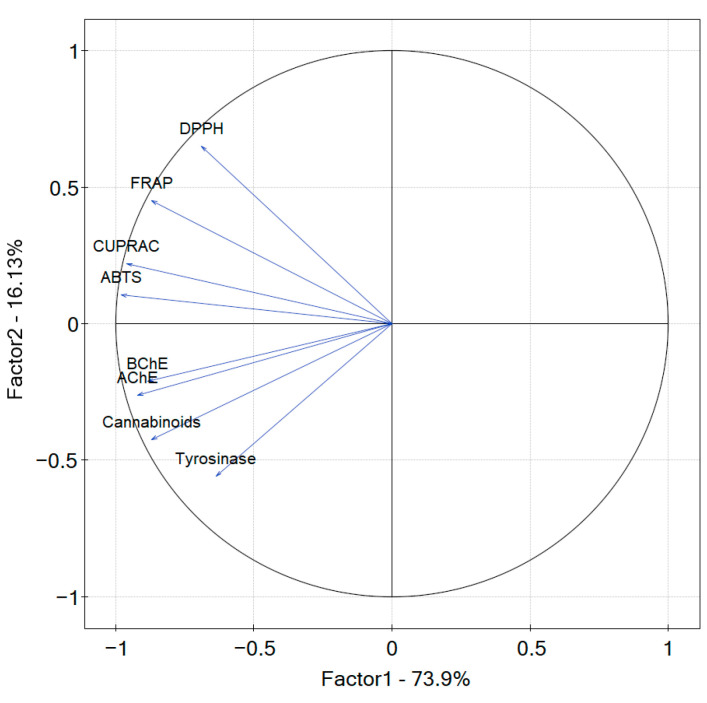
Contributions of variables (DPPH, ABTS, CUPRAC, FRAP, AChE, BChE, tyrosinase) to PCs.

Factor 1 (principal component PC1) accounted for approximately 73.9% of the observed variation in the samples, whereas factor 2 (principal component PC2) explained approximately 16.13% of the variation (Figure 8). The ABTS, CUPRAC, FRAP, AChE, and BChE values strongly correlate with PC1 negatively, whilst DPPH and tyrosinase correlate with PC1 negatively to a smaller extent. However, the higher values of the coordinates of the vector’s endpoint are observed more for the first component than for the second one. There is a strong relation between the antioxidant (DPPH, ABTS, CUPRAC, FRAP) values and the potential to inhibit the enzymes AChE, BChE, and tyrosinase. The sum of the eight cannabinoids relates with all of the studied biological activity directions as the angle between the vectors is less than 90°. However, the strongest relation is noticed between the sum of the cannabinoids and enzymatic inhibitory potential and the weakest with DPPH. 

Three antioxidant assays: ABTS, FRAP, and CUPRAC, showed a strong correlation with some of the cannabinoids and the strongest with CBDA—0.941, 0.774, and 0.885, respectively [102]. AChE and BChE showed the highest correlation with CBD (0.858, 0.845, respectively) and CBDA content (0.860, 0.820, respectively). The tyrosinase inhibitory potential was the closest correlated to CBG and CBN content—r-Pearson correlation coefficient 0.809 and 0.787, respectively. When analyzing the data for the leaves and inflorescences separately, the r-Pearson correlation coefficients vary, as the overall composition and content of compounds that were not determined, such as terpenes, terpenoids, and flavonoids, are different. The r-Pearson correlation coefficient analysis confirmed that DPPH has the weakest correlation with the cannabinoid content, with the greatest value of 0.533 provided for CBDA. 

The Tygra inflorescences extract (b) exhibited the highest antioxidant potential among the tested extracts. The most effective inhibition was observed in the case of AChE and BChE by the Henola inflorescences extract (b). The inhibition of tyrosinase was most pronounced in the case of the Białobrzeskie inflorescences extract (b). As the result of multidimensional comparative analysis, the Henola inflorescences (b) extract was determined as one with substantial neuroprotective potential, which comprises antioxidant activity and the ability to inhibit enzymes related to neurodegeneration.

Patients’ preferences have undergone significant changes, reflecting a growing demand for natural and alternative remedies [103,104]. Hemp extracts have garnered substantial attention in recent years due to their diverse therapeutic potential, including anti-inflammatory, pain-relieving, and antiepileptic properties [105]. These benefits are attributed to the presence of various natural compounds, such as cannabinoids, flavonoids, and terpenes. Current research primarily focuses on exploring antioxidant properties and the ability to inhibit enzymes associated with neurodegenerative diseases, which may have preventive applications. The increased demand for hemp-derived extracts has led to expanded cultivation, benefiting the agricultural sector. Importantly, hemp is considered an environmentally sustainable crop because it requires less water and fewer pesticides compared to other crops [106]. Moreover, the environmentally friendly extraction methods used for cannabis align with the growing emphasis on sustainability and eco-conscious consumer choices [107]. Nevertheless, it is crucial to acknowledge that depending on the specific product, integrating cannabis into one’s daily life may still incur significant associated costs [108,109,110]. The Henola variety, with its low THC content, presents an intriguing opportunity for cultivation in many countries and states where strains with higher THC levels face legal restrictions [111,112,113,114]. This characteristic provides legal compliance and makes it attractive for disease prevention without causing psychoactive effects [115]. Utilizing the entire hemp plant can enhance returns and justify the sustainability and cost-effectiveness of Henola cultivation. The hemp extract market is dynamic, featuring various product categories such as CBD oils, tinctures, edibles, and topicals [116,117,118]. Henola inflorescence extract can be employed in various forms, including oils, and may be suitable for oral administration following preformulation studies.

## 4. Conclusions

All the tested varieties of hemp (Białobrzeskie, Henola, and Tygra) showed the potential for antioxidant and neuroprotective properties, expressed by the possibility of scavenging radicals, reducing ferric and cupric ions, and inhibiting esterases and tyrosinase, but the greatest neuroprotective effect was found in extracts from Henola hemp inflorescences obtained as a result of extraction with carbon dioxide at 50 °C under and under 6000 PSI. Due to the promising results, the Henola variety holds the highest potential for effective use in neurodegeneration prevention, while also contributing to the reduction in associated agricultural waste.

Bearing in mind all the advantages of supercritical carbon dioxide extraction in the face of the confirmed neuroprotective effect of the leaves and inflorescences of the tested hemp varieties, this method of obtaining preparations/semi-finished products should be recommended as the most optimal and safe in the development of products containing hemp varieties such as Białobrzeskie, Henola, and Tygra. 

## Figures and Tables

**Table 1:** The parameters of supercritical carbon dioxide extraction. (L—leaves, I—inflorescences).

Name	Plant Material	Solvent	Temp.	Pressure
Białobrzeskie L (a)	Leaves	Supercritical CO_2_	50 °C	2000 PSI
Białobrzeskie L (b)				6000 PSI
Henola L (a)				2000 PSI
Henola L (b)				6000 PSI
Tygra L (a)				2000 PSI
Tygra L (b)				6000 PSI
Białobrzeskie I (a)	Inflorescences	Supercritical CO_2_	50 °C	2000 PSI
Białobrzeskie I (b)				6000 PSI
Henola I (a)				2000 PSI
Henola I (b)				6000 PSI
Tygra I (a)				2000 PSI
Tygra I (b)				6000 PSI

**Table 2:** The retention time of cannabinoids.

Cannabinoid	Retention Time (min)
CBD	5.84
CBDA	6.42
CBG	6.82
CBN	8.72
CBGA	9.22
Δ^9^-THC	10.27
CBC	14.57
THCA	16.31

**Table 3:** The content of cannabinoids present in Białobrzeskie, Henola, and Tygra leaves (L) and inflorescences (I) extracts obtained under 2000 PSI (a) and 6000 PSI (b): CBD (cannabidiol), CBDA (cannabidioloc acid), CBG (cannabigerol), CBN (cannabinol), CBGA (cannabigerolic acid), Δ9-THC ((-)-delta 9-tetrahydrocannabinol), CBC (cannabichromene), THCA (tetrahydrocannabinolic acid) presented as μg cannabinoid/g plant material.

Extract	CBD	CBDA	CBG	CBN	CBGA	THC	CBC	THCA
	μg/g Plant Material							
Białobrzeskie L (a)	90.92 ± 1.65	77.25 ± 1.00	N/D	3.66 ± 0.12	N/D	24.39 ± 0.84	8.18 ± 0.19	3.13 ± 0.07
Białobrzeskie L (b)	207.6 ± 5.5	109.6 ± 1.4	3.75 ± 0.08	3.51 ± 0.15	1.02 ± 0.05	13.55 ± 0.17	3.81 ± 0.11	6.29 ± 0.17
Henola L (a)	201.7 ± 6.3	42.84 ± 0.12	3.72 ± 0.05	N/D	N/D	7.83 ± 0.21	7.18 ± 0.09	N/D
Henola L (b)	206.2 ± 2.7	134.3 ± 0.2	5.02 ± 0.17	2.07 ± 0.02	1.28 ± 0.08	9.85 ± 0.42	9.28 ± 0.26	2.39 ± 0.08
Tygra L (a)	278.8 ± 7.3	90.59 ± 0.99	6.44 ± 0.19	3.57 ± 0.09	0.83 ± 0.04	31.75 ± 0.04	16.42 ± 0.58	6.67 ± 0.15
Tygra L (b)	264.1 ± 3.1	242.6 ± 5.1	6.02 ± 0.08	3.86 ± 0.05	1.73 ± 0.09	32.48 ± 0.18	16.92 ± 0.28	18.71 ± 0.77
Białobrzeskie I (a)	2378.4 ± 17.1	280.4 ± 5.6	60.99 ± 0.58	34.88 ± 1.60	3.47 ± 0.14	391.9 ± 5.6	129.9 ± 5.4	34.81 ± 0.34
Białobrzeskie I (b)	4510.2 ± 53.1	1912.2 ± 15.8	224.8 ± 2.0	107.7 ± 5.4	89.74 ± 4.23	998.4 ± 18.8	310.2 ± 12.9	303.0 ± 3.9
Henola I (a)	3436.7 ± 75.6	403.4 ± 13.6	224.8 ± 1.5	10.91 ± 0.14	N/D	119.4 ± 6.0	130.0 ± 2.0	7.47 ± 0.08
Henola I (b)	6027.4 ± 55.3	2078.7 ± 44.3	109.1 ± 1.5	40.23 ± 0.12	22.46 ± 0.59	280.0 ± 9.7	251.684 ± 6.858	57.75 ± 1.33
Tygra I (a)	4659.6 ± 144.9	507.6 ± 15.9	109.1 ± 2.5	21.19 ± 0.70	N/D	175.1 ± 2.3	214.8 ± 4.0	6.53 ± 0.07
Tygra I (b)	6248.5 ± 154.9	2558.0 ± 107.8	125.9 ± 3.0	43.18 ± 0.60	33.38 ± 1.05	282.5 ± 4.5	300.7 ± 1.3	38.25 ± 1.22

## Data Availability

Data are available in a publicly accessible repository.

## Abbreviations

ABTS	2,2′-azino-bis(3-ethylbenzothiazoline-6-sulfonic acid)
AChE	Acetylcholinesterase
ANOVA	one-way analysis of variance
ATCI	acetylthiocholine iodide
AzAE	azelaic acid equivalent
BChE	butyrylcholinesterase
BTCI	butyrylthiocholine iodide
CBC	cannabichromene
CBD	cannabidiol
CBDA	cannabidiolic acid
CBG	cannabigerol
CBGA	cannabigerolic acid
CBN	cannabichromene
CUPRAC	cupric reducing antioxidant capacity
DAD	diode array detector
DPPH	2,2-diphenyl-1-picrylhydrazyl
DTNB	5,5′-dithio-bis-(2-nitrobenzoic) acid
FRAP	ferric reducing antioxidant power
GALAE	galantamine equivalent
HPLC	high-performance liquid chromatography
I	inflorescences
L	leaves
MCA	multidimensional comparative analysis
PC	principal component
PCA	principal component analysis
ROS	reactive oxygen species
SFE	supercritical fluid extraction
THC	Δ-9-tetrahydrocannabinol
THCA	Δ-9-tetrahydrocannabinolic acid
TNB	3-carboxy-4-nitrothiolate
TPTZ	2,4,6-tris(2-pyridyl)-1,3,5-triazine
